# The multiple actions of dipeptidyl peptidase 4 (DPP-4) and its pharmacological inhibition on bone metabolism: a review

**DOI:** 10.1186/s13098-024-01412-x

**Published:** 2024-07-25

**Authors:** L. M. Pechmann, F. I. Pinheiro, V. F. C. Andrade, C. A. Moreira

**Affiliations:** 1 Universidade Federal do Paraná, Setor de Ciências da Saúde, Endocrine Division (SEMPR), Centro de Diabetes Curitiba, Academic Research Center Pro Renal Institute, Curitiba, Brazil; 2 Biotechnology at Universidade Potiguar and Discipline of Ophthalmology at the Federal University of Rio Grande do Norte (UFRN), Natal, Brazil; 3 Academic Research Center Pro Renal Institute, Endocrine Division, Hospital de Cínicas da Universidade Federal do Paraná (SEMPR), Curitiba, Brazil; 4 Academic Research Center Pro Renal Institute, Endocrine Division, Hospital de Clinicas da Universidade Federal do Paraná ( SEMPR), Curitiba, Brazil

**Keywords:** Dipeptidyl peptidase 4, Dipeptidyl-peptidase IV inhibitors, Bone diseases, Metabolic, Osteoclasts, Incretins, Osteoporosis, Fractures, Bone, Bone resorption, Osteogenesis

## Abstract

**Background:**

Dipeptidyl peptidase 4 (DPP-4) plays a crucial role in breaking down various substrates. It also has effects on the insulin signaling pathway, contributing to insulin resistance, and involvement in inflammatory processes like obesity and type 2 diabetes mellitus. Emerging effects of DPP-4 on bone metabolism include an inverse relationship between DPP-4 activity levels and bone mineral density, along with an increased risk of fractures.

**Main body:**

The influence of DPP-4 on bone metabolism occurs through two axes. The entero-endocrine-osseous axis involves gastrointestinal substrates for DPP-4, including glucose-dependent insulinotropic polypeptide (GIP) and glucagon-like peptides 1 (GLP-1) and 2 (GLP-2). Studies suggest that supraphysiological doses of exogenous GLP-2 has a significant inhibitory effect on bone resorption, however the specific mechanism by which GLP-2 influences bone metabolism remains unknown. Of these, GIP stands out for its role in bone formation. Other gastrointestinal DPP-4 substrates are pancreatic peptide YY and neuropeptide Y—both bind to the same receptors and appear to increase bone resorption and decrease bone formation. Adipokines (*e.g.*, leptin and adiponectin) are regulated by DPP-4 and may influence bone remodeling and energy metabolism in a paracrine manner. The pancreatic-endocrine-osseous axis involves a potential link between DPP-4, bone, and energy metabolism through the receptor activator of nuclear factor kappa B ligand (RANKL), which induces DPP-4 expression in osteoclasts, leading to decreased GLP-1 levels and increased blood glucose levels. Inhibitors of DPP-4 participate in the pancreatic-endocrine-osseous axis by increasing endogenous GLP-1. In addition to their glycemic effects, DPP-4 inhibitors have the potential to decrease bone resorption, increase bone formation, and reduce the incidence of osteoporosis and fractures. Still, many questions on the interactions between DPP-4 and bone remain unanswered, particularly regarding the effects of DPP-4 inhibition on the skeleton of older individuals.

**Conclusion:**

The elucidation of the intricate interactions and impact of DPP-4 on bone is paramount for a proper understanding of the body's mechanisms in regulating bone homeostasis and responses to internal stimuli. This understanding bears significant implications in the investigation of conditions like osteoporosis, in which disruptions to these signaling pathways occur. Further research is essential to uncover the full extent of DPP-4's effects on bone metabolism and energy regulation, paving the way for novel therapeutic interventions targeting these pathways, particularly in older individuals.

## Introduction

Dipeptidyl peptidase 4 (DPP-4) is a serine peptidase found in the form of a surface protein anchored to the cell membrane or soluble in plasma. This enzyme has been increasingly recognized for its multifaceted role in various physiological processes, extending beyond its initially known functions in insulin signaling, inflammation, and energy metabolism [1]. Various cells in the bone microenvironment secrete DPP-4, including osteoclasts, bone marrow adipose tissue, and immune cells [2]. The association of DPP-4 with osteoclasts suggests a stimulatory action of this enzyme on bone resorption [3, 4], potentially affecting bone mineral density (BMD) and fracture risk and identifying DPP-4 activity as a potential marker of altered bone metabolism [5–10]. In fact, studies have revealed an inverse correlation between DPP-4 activity levels and BMD, suggesting a likely role of DPP-4 in bone homeostasis [5–8, 10]. Additionally, individuals with increased DPP-4 activity may have an elevated risk of fractures, implicating DPP-4 in skeletal fragility [5, 7, 10]. These findings highlight the importance of understanding the intricate interplay between DPP-4, bone metabolism, and systemic health [9, 11–19].

Recently, DPP-4 emerged as an adipokine/hepatokine with potential connections to skeletal muscle function and BMD [20]. Indeed, DPP-4 acts as a receptor or costimulatory protein in immunomodulatory signaling processes in various immune cells such as CD8 + and CD4 + T cells, B cells, and macrophages, and hydrolyzes different sites of chemokines and interleukins that are part of bone remodeling. The effects of DPP-4 on bone health are underscored by its effects in generating proinflammatory cytokines such as interleukin-6 (IL-6) and tumor necrosis factor-alpha (TNF-α), contributing to inflammatory processes mediated by adipose tissue macrophages, which are implicated in conditions ranging from obesity to osteoporosis [21]. In fact, mice with hepatocyte-specific DPP-4 knockdown have a significant reduction in serum DPP-4 activity and reduced adipose tissue inflammation, insulin resistance, and glucose intolerance [20]. Expression of DPP-4 is substantially dysregulated in a variety of disease states, including inflammation, cancer, obesity, and diabetes [22]. This suggests that DPP-4 inhibitors, which are commonly used for treating type 2 diabetes mellitus (T2DM), may offer therapeutic benefits beyond glycemic control, potentially mitigating bone resorption and reducing fracture risk [23]. Clinical studies investigating the effects of DPP-4 inhibitors have yielded promising results, indicating improvements in bone density and a potential decrease in fracture incidence. However, conflicting findings and gaps in understanding persist, calling for further research into the mechanisms underlying the influence of DPP-4 on bone metabolism. Of particular interest are the paracrine effects of adipokines and gastrointestinal substrates regulated by DPP-4, such as leptin, adiponectin, pancreatic peptide YY (PYY), and glucagon-like peptide 1 (GLP-1) and glucagon-like peptide 2 (GLP-2), which may mediate the crosstalk between bone remodeling and energy metabolism [2, 24]**.**

In summary, elucidating the bone effects of DPP-4 holds significant implications for both clinical practice and basic research. By unraveling the complex interconnections between DPP-4, energy metabolism, and bone health, we can uncover valuable insight to guide the development of innovative treatments for conditions ranging from T2DM to osteoporosis.

## Molecular structure of DPP-4

Initially described by Hopsu-Havu & Glenner in 1966 [25], DPP-4 is a dimeric 240-kDa glycoprotein composed of two 120-kDa subunits and encoded by a gene located in chromosome 2q24 [25].

**Fig. 1:**
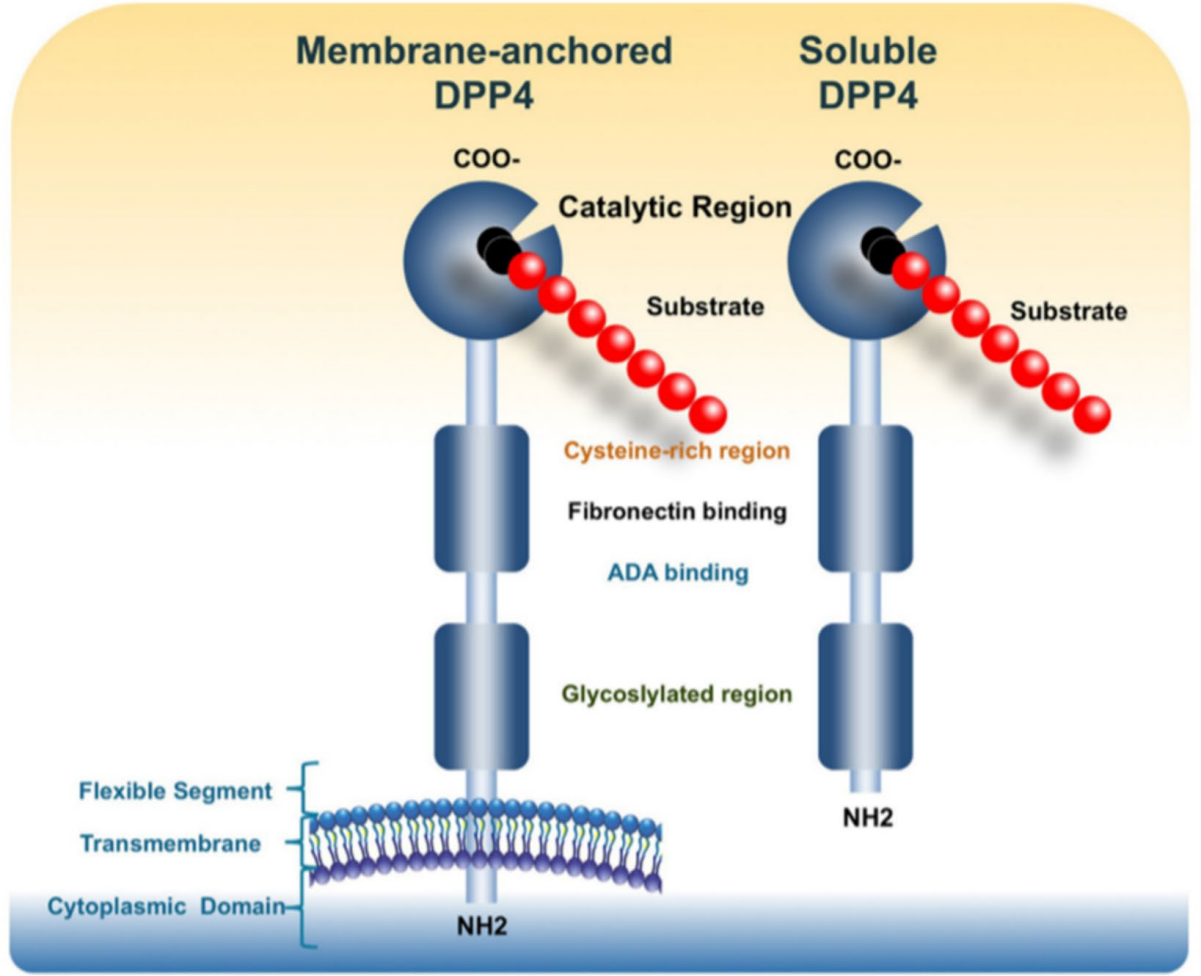
Schematic representation of the dipeptidyl peptidase 4 (DPP-4) monomer bound to the membrane and the soluble DPP-4. Schematic representation of the dipeptidyl peptidase 4 (DPP-4) monomer bound to the membrane and to soluble DPP-4. Catalytically active DPP-4 is released from the plasma membrane, producing a soluble circulating form (*i.e.*, soluble DPP-4, which contains 727 amino acids). The soluble DPP-4 lacks the intracellular and transmembrane domains and accounts for a substantial proportion of DPP-4 activity in human serum. Both membrane-bound and circulating soluble DPP-4 share many domains, including the glycosylated region (residues 101–535, specific residues 85, 92, 150), ADA binding domain (340–343), fibronectin binding domain (468–479), cysteine-rich domain (351–506, disulfide bonds are formed from 385–394, 444–472, and 649–762), and the catalytic domain (507–766, including residues composing the catalytic active site 630, 708, and 740). Adapted from Mulvihill et al. Endocrine Reviews, December 2014, 35(6):992–1019 (20). Reproduced with permission from Oxford University Press and Copyright Clearance Center (License number 570255101696)

Structurally, DPP-4 is formed by three domains: short cytoplasmic, transmembrane, and extracellular (Fig. 1). The extracellular domain is further subdivided into three regions, *i.e.*, glycosylated, cysteine-rich, and catalytic (or C-terminal, [22]). The glycosylated and cysteine-rich regions are involved in nonenzymatic functions of the enzyme and interact with other proteins (*e.g.*, adenosine deaminase (ADA), caveolin-1, streptokinase, and plasminogen) and components of the extracellular matrix (*e.g.*, collagen and fibronectin.) The best-studied interaction in this regard is certainly the binding of DPP-4 and ADA. Furthermore, ADA activity is elevated in patients with T2DM and may serve as a marker of inflammation and obesity. Via interaction with CD45, the complex of ADA and DPP-4 enhances T-cell activation [22, 26]. A flexible segment in DPP-4 connects the transmembrane domain to the extracellular domain and is the target of shedding, a process in which the enzyme is cleaved and released into circulation [22]. The extracellular portion released as soluble DPP-4 is found in plasma and biological fluids and can be quantified both in terms of activity and concentration [9]. In addition to the soluble isoform, DPP-4 presents an enzymatic form, each with different roles in influencing various physiological processes controlling inflammation and glucose homeostasis. While enzymatic DPP-4 may be more closely related to obesity-associated inflammation and glucose regulation, soluble DPP-4 may have a distinct role that is not associated with inflammation. Overall, 90–95% of serum DPP-4 activity is related to soluble DPP-4 levels [27–31].

A study has analyzed how plasma DPP-4 activity and levels of soluble DPP-4 correlate with inflammatory markers (C-reactive protein [CRP], IL-6, TNF-α, and monocyte chemoattractant protein-1 [MCP-1]) in a subset of patients with T2DM treated with sitagliptin for 12 months as part of the Trial Evaluating Cardiovascular Outcomes with Sitagliptin (TECOS, 26). As expected, treatment with sitagliptin led to a significant reduction in plasma DPP-4 activity at 12 months, but the levels of soluble DPP-4 and inflammatory markers remained unchanged [25]. These findings indicate a dissociation in the modulation of DPP-4-related parameters and inflammatory biomarkers in humans [32, 33].

## Mechanisms of DPP-4 action

Widely distributed, DPP-4 is present on the surface of various cells, including adipocytes and liver, kidney, intestine, endothelial, and immune cells [34]. Initial studies had indicated DPP-4 to be an adipokine due to its release from the adipocyte membrane through the action of matrix metallopeptidase 9 (MMP9), resulting in the release of the soluble DPP-4 form in the circulation [35]. Subsequently, Lamers et al. described a strong correlation between soluble DPP-4 and adipocyte size, suggesting an important link between DPP-4 and obesity [34].

Recent studies have uncovered increased DPP-4 expression and secretion from hepatocytes in obese mice, with a DPP-4 expression and activity much higher in the liver than in adipose tissue, indicating its emerging role as a hepatokine in the interplay between hepatocytes and adipocytes [4]. Conversely, selective loss of adipocyte DPP-4 enhances hepatic insulin sensitivity and reduces inflammation, with no effects on glucose tolerance [4]. These findings have set the stage for Varin et al. to explore the roles of DPP-4. These authors discussed the presence of circulating soluble DPP-4—a DPP-4 form distinct from the enzymatic DPP-4—and proposed that while enzymatic DPP-4 may be linked to obesity-associated inflammation and glucose regulation, soluble DPP-4 may have separate functions unrelated to inflammation [32]. Overall, these studies underscore the intricate relationships between DPP-4, glucose regulation, obesity, and inflammation, highlighting its complexity and interactions with bone metabolism, along with its regulatory mechanisms, suggesting potential therapeutic implications [4, 32, 36].

Several effects have been associated with DPP-4, including degradation of various substrates (such as incretins, neuropeptides, and cytokines) and involvement with inflammatory processes (including cancer, obesity, and T2DM, 20, 37). Additionally, DPP-4 exhibits an inverse correlation with BMD, suggesting a potential connection with osteoporosis [5–8, 10].

**Fig. 2:**
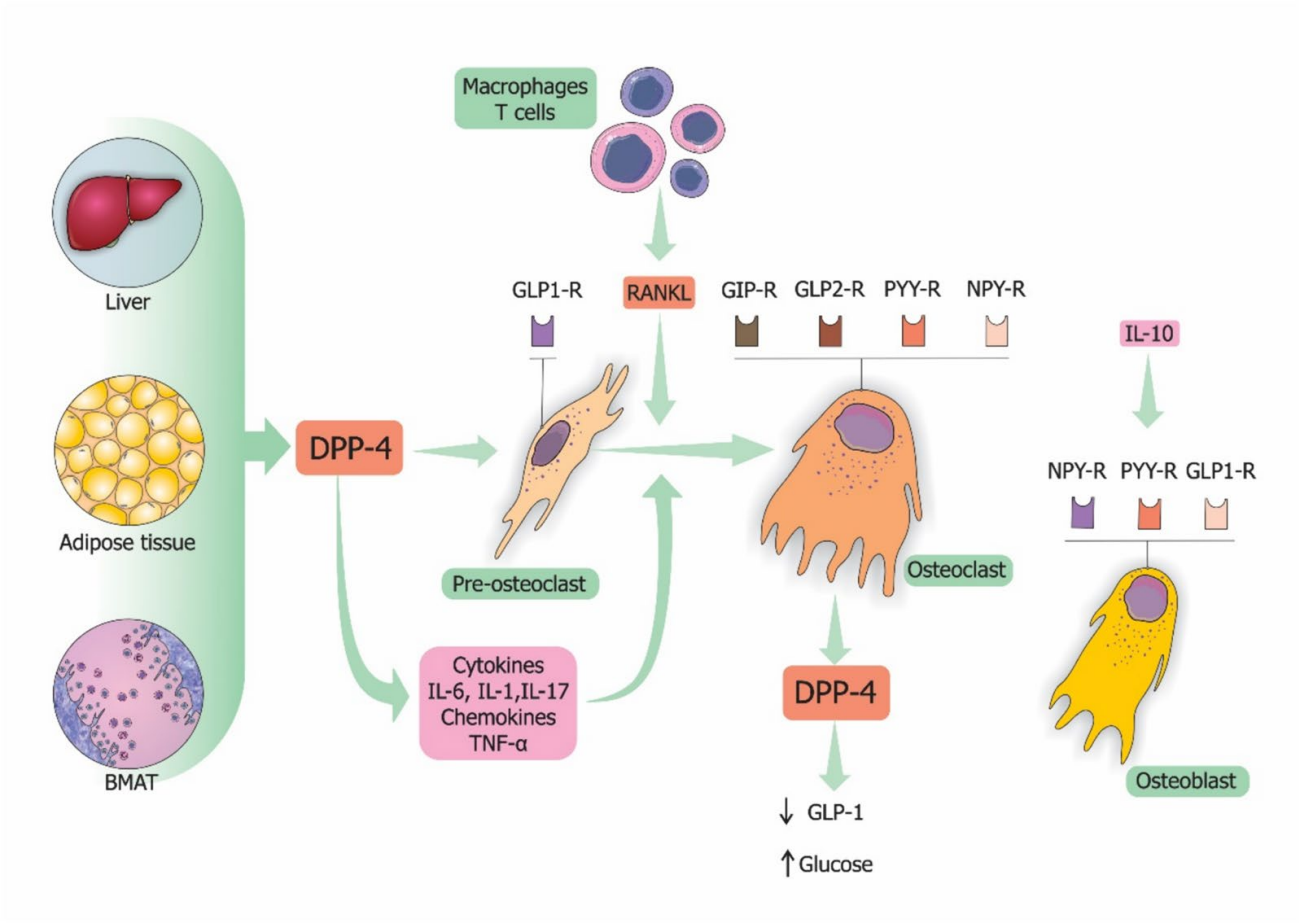
Potential mechanisms of action of dipeptidyl peptidase 4 on bone metabolism*. BMAT, bone marrow adipose tissue; DPP-4, dipeptidyl peptidase 4; GLP1-R, receptor for glucagon-like peptide 1 (GLP-1); GIPR, receptor for glucose-dependent insulinotropic polypeptide (GIP); IL, interleukin; PYR, receptor for peptide YY; NPYR, receptor for neuropeptide Y; RANKL, receptor activator of nuclear factor-kappa B ligand, TNF-α, tumor necrosis factor-alpha. Complex roles of DPP-4 in classical enzymatic and nonenzymatic functions of bone metabolism. Bone marrow mesenchymal cells, liver, and adipose tissue produce DPP-4, while RANKL induces the expression of DPP-4 by osteoclasts, leading to decreased GLP-1 levels and increased blood glucose levels. Further, DPP-4 cleaves various sites on chemokines, interleukins, and other cytokines that participate actively in bone remodeling. Potentially, DPP-4 exerts indirect regulation of bone remodeling by interacting with multiple peptide substrates on bone cells, including GLP-1, glucagon-like peptide-2 (GLP-2), GIP, NPY, and PYY

Weivoda et al. presented compelling evidence indicating the occurrence of a pancreatic-endocrine-bone axis governing fuel metabolism in humans [3]. Using RNA sequencing of bone biopsies from patients treated with denosumab compared with placebo, the authors observed a down-regulation of skeletal DPP-4 expression with denosumab [3]. Further investigation using in situ hybridization revealed DPP-4 expression in the osteoclast lineage. Additionally, RANKL emerged as a potential link between DPP-4 and bone-energy metabolism, as it induced DPP-4 expression in osteoclasts, leading to decreased GLP-1 levels and increased blood glucose (Fig. 2).

Patients with T2DM treated with denosumab exhibit lower glycated hemoglobin levels compared with those treated with bisphosphonates or calcium and vitamin D supplementation, highlighting the role of the RANK-RANKL system and implicating DPP-4 as a potential mediator between bone remodeling and energy metabolism [3]. These findings underscore the multifaceted roles of DPP-4, not only as an osteoclast-derived protein but also as a connector between bone remodeling and energy metabolism, with significant implications for the pancreatic-endocrine-bone axis [3, 36, 37].

The modulation of glucose metabolism is one of the most relevant effects of DPP-4 in clinical practice [23]. The idea of regulating glucose levels through DPP-4 inhibition was initially conceived 25 years ago, paving the way for the development of different DPP-4 inhibitors and their widespread clinical utilization [38]. Extensive clinical experience has been reported using these medications in a wide spectrum of patients with T2DM and concomitant cardiovascular disease, chronic kidney disease, or obesity, among others. These medications potently and selectively inhibit the enzymatic activity of DPP-4, enhancing the effectiveness of GLP-1 and glucose-dependent insulinotropic polypeptide (GIP), which are the primary incretins (endogenous glucoregulatory peptides, [39–41])**.**

### Entero-endocrine-osseous *axis*: gastrointestinal hormones as substrates for DPP-4

The initial observation that patients receiving long-term parenteral nutrition develop osteoporosis and osteomalacia raised suspicion about the lack of stimulation for the secretion of incretin hormones in this mode of nutrition and a potential connection between these hormones and bone metabolism [42]. This has led to the exploration of a potential connection between incretin hormones and bone tissue, referred to as the entero-endocrine-osseous axis. Further evidence supporting this hypothesis comes from the typical decrease in bone turnover observed after oral glucose intake, which is inhibited by infusion of octreotide, a somatostatin analog that suppresses the secretion of gastrointestinal and pancreatic peptides [43]. These findings suggest that the gut plays a crucial role in postprandial bone remodeling [42, 43].

The incretin hormones GIP and GLP-1 are important substrates for DPP-4 action, while increased DPP-4 activity is associated with lower levels of GIP and GLP-1 [3]. Notably, GIP is secreted by the enteroendocrine K-cells that are present in high density in the duodenum and upper jejunum, while GLP-1-producing cells of the intestine are mainly positioned in the distal parts of the gut [44]. Serum levels of GIP and GLP-1 increase approximately five times after a meal [45]. The breakdown of GIP and GLP-1 by DPP-4 occurs approximately 4 min after these hormones enter the circulation. Studies show that these peptides have a favorable effect on bone metabolism, although these effects are still poorly understood [46].

#### GIP

Similar to the other two gut-derived hormones (GLP-1 and GLP-2), GIP influences bone remodeling as part of the entero-endocrine-osseous axis. Receptors for GIP are expressed in osteoblasts and bone marrow cells [47]. Additionally, GIP is expressed in osteoclasts, and its binding to the receptor inhibits bone resorption [21]. Studies in animals with GIP knockout genes have shown different results depending on the deleted exon. In general, GIP knockout leads to decreased bone formation parameters (*e.g.*, BMD, bone mineral content, trabecular bone volume, alkaline phosphatase, and osteocalcin) and increased resorptive parameters (*e.g.*, greater number of osteoclasts and increased urinary elimination of the resorption marker deoxypyridinoline, [48]). Another study in a GIP receptor knockout model showed decreased bone strength and cortical thickness and increased bone resorption—but paradoxically, an increased number of osteoblasts and a reduced number of osteoclasts [49].

In humans and rodents, GIP infusion results in decreased levels of cross-linked C-terminal telopeptide of type I collagen (CTX-1) and increased levels of procollagen type I N-terminal propeptide (P1NP), regardless of whether blood glucose levels are normal or elevated [50–52]**.** Observational studies have shown that GIP receptor mutations lead to decreased receptor signaling, which results in lower BMD and increased risk of fractures [50]. Additionally, GIP may stimulate bone formation, indicating a possible separation between the processes of bone resorption and formation [47].

Some studies involving healthy subjects reported that endogenous GIP contributes to up to 25% of the suppression of bone resorption after a meal, while it found that endogenous GLP-1 has no impact on postprandial bone homeostasis [53, 54]**.**

**Fig. 3:**
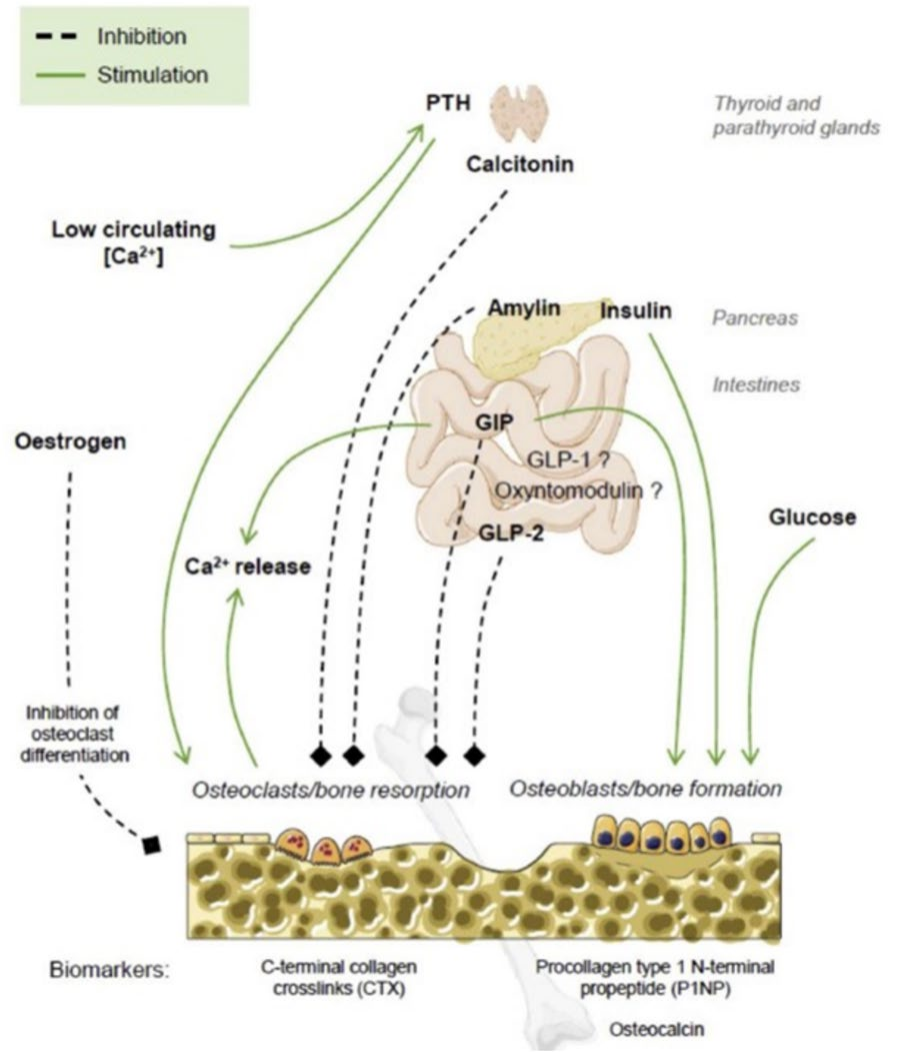
Entero endocrine-osseous axis The entero-endocrine-osseous axis. Lower serum calcium levels stimulate the parathyroid release of PTH, which increases bone reabsorption with release of calcium into the circulation. Thyroid C cells present receptors for GLP-1, as demonstrated in preclinical studies, and stimulation of calcitonin production inhibits osteoclastic activity. The contributions of endogenous GIP to postprandial bone homeostasis are as follows: endogenous GIP contributes to the postprandial suppression of bone resorption in humans and stimulates bone formation through stimulation of osteoblasts [47]. Both GIP and GLP‐2 receptors are expressed in parathyroid tissue, and the effect of GLP‐2 on bone turnover seems to depend on changes in PTH levels and may be mediated through GLP‐2 receptor in the parathyroid gland. Effects of GIP on bone turnover may be mediated directly via GIP receptor expressed in osteoblasts and osteoclasts, which may occur independently from PTH [47]. SOURCE: Adapted from Stensen et al. The enterosseous axis and its relationship with thyroid C cells and PTH. Copyright provided by Elsevier and Copyright Clearance Center. License Number 5702571099338. Abbreviations: GIP, glucose-dependent insulinotropic polypeptide; GLP-1, glucagon-like peptide 1; GLP-2, glucagon-like peptide 2; CTX, carboxy-terminal type 1 collagen crosslinks; P1NP, procollagen type 1 amino-terminal propeptide

In summary, GIP influences bone remodeling through an entero-endocrine-osseous axis and plays a role in coordinating optimal bone turnover in response to food intake, mainly during the day. Both exogenous and endogenous GIP decrease bone resorption in humans [50]. This suggests that the GIP receptor could be a potential target for the prevention and treatment of osteoporosis [50], Fig. 3).

#### GLP-1

Multiple studies in rodents have established the role of GLP-1 in bone metabolism. Indeed, mice osteoblasts, osteocytes, and osteoclasts have been shown to express GLP-1 receptors [21]. The primary GLP-1 action in rodents' bone is to promote bone formation by stimulating osteoblasts through the regulation of runt-related transcription factor 2 (RUNX2), alkaline phosphatase, collagen type 1, and osteocalcin [55]. Additionally, GLP-1 acts directly and indirectly on the Wnt/β-catenin pathway by reducing the mRNA levels of sclerostin, a known inhibitor of bone formation [55]. In rodents, stimulation of GLP-1 receptors in thyroid C cells promotes the secretion of calcitonin. This hormone, in turn, inhibits osteoclastic activity, which decreases the release of calcium from the bone into the bloodstream, leading to decreased bone resorption [56, 57]. Prolonged administration of high-dose liraglutide (a GLP-1 receptor agonist) to monkeys did not result in calcitonin secretion or C-cell hyperplasia. This indicates marked differences in the effects of GLP-1 on bone metabolism between different mammalian species [56, 58].

In summary, GLP-1 has positive effects on bone strength and quality in rats and protects against bone loss. It increases bone formation parameters and decreases bone resorption parameters. These findings suggest an essential role for endogenous GLP-1 receptor signaling in the control of bone resorption. In rodents, this effect likely occurs through a calcitonin-dependent pathway since GLP-1 does not appear to have a direct effect on osteoblasts and osteoclasts in vitro [56].

Findings from human studies focused on GLP-1 actions on bone are inconsistent. Agonists of the GLP-1 receptor have been shown to increase levels of markers of bone formation (osteocalcin and procollagen type 1 N-terminal propeptide [P1NP]) and protect against loss of bone mineral content in obese women after weight loss while having no effect on plasma CTX-1 concentrations [59, 60]. In a retrospective cohort study, patients with T2DM and concomitant osteoporosis or osteopenia who used DPP-4 inhibitors and no antiosteoporotic medications were divided into two groups: those who switched to a GLP-1 receptor agonist and those who continued on a DPP-4 inhibitor [61]. The authors compared changes in glycemic control and BMD with and without conversion from DPP-4 inhibitor to GLP-1 receptor agonist for 3 years and observed that patients who switched to the latter had greater decline in lumbar BMD than controls regardless of weight loss [12, 61].

A meta-analysis of randomized clinical trials evaluating the use of GLP-1 receptor agonists and the occurrence of bone fractures in patients with T2DM observed that these medications did not reduce the incidence of fractures compared with other antidiabetic medications [62]. In contrast, another meta-analysis observed that the risk of fractures was reduced with liraglutide but increased with exenatide (also a GLP-1 receptor agonist, [63]. In a systematic review and network meta-analysis, Zhang et al. found benefits from GLP-1 receptor agonists in terms of fracture risk [13]. Notably, these authors included in their analysis only randomized controlled trials with a duration ≥ 52 weeks considering that interventions shorter than that were unlikely to affect the fracture risk [13].

The conclusions of most clinical studies on GLP-1 effects are insufficient to provide strong evidence. Although GLP-1 receptor agonists show benefits in animal models, limited clinical data preclude researchers from drawing confident conclusions [64, 65]. Discrepant findings in humans may be due to the short duration of the studies (on average 35 weeks) and the fact that fractures have not been considered a primary outcome in the studies, but rather, an adverse event [56], (Table 1).

**Table 1:** Actions of dipeptidyl peptidase 4 (DPP-4) substrates on bone remodeling

	Bone formation parameters	Bone reabsorption parameters
GLP-1		
Preclinical studies [55, 56, 58]	Increase	Decrease/stimulation of calcitonin secretion and inhibition of osteoclastic activity. No effect on CTX-1
Clinical studies [12, 13, 56, 61, 62]	Controversial (varies with type of study)	No effect
GIP		
Preclinical studies [47–49, 52]	Increase	Decrease in CTX-1
Clinical studies (52[51, 53, 54]	Increase	Decrease
GLP-2		
Clinical studies [70]	No effect	Decrease
GIP + GLP-1		
Clinical studies [71]	Increase	Decrease
LEPTIN		
Preclinical studies*[102, 106, 108]	Increase (cortical bone)	Increase (trabecular bone)
Clinical studies [94, 102–106]	Potential increase	N/A
ADIPONECTIN		
Preclinical studies [86, 87, 92]	Increase	Decrease
Clinical studies [85, 88–91, 93–96]	Discrepant results	Probable increase
NPY**		
Preclinical studies [73, 74, 76, 77, 79]	Decrease	Increase
Clinical studies [78]	Decrease	Increase
PYY		
Preclinical studies [77]	Increase	Decrease
Clinical studies [80–82]	Decrease	Increase

^*^Central (intraventricular) administration of leptin in ob/ob mice. ** Via Y1 and Y2 receptors. *CTX-1* cross-linked C-terminal telopeptide of type I collagen, *GIP* glucose-dependent insulinotropic polypeptide, *GLP-1* glucagon-like peptide 1, *GLP-2* glucagon-like peptide 2; *N/A* not applicable, *NPY* neuropeptide Y, *PYY* peptide YY

In summary, human studies analyzing the effects of GLP-1 receptor agonists on bone show inconsistent results. While these analogs may protect against bone mineral content loss and increase bone formation indicators, they show no effect on plasma CTX-1 concentrations. Meta-analyses on GLP-1 receptor agonists and fracture occurrence have yielded conflicting results, possibly due to short study durations and fractures not being primary outcomes. Limited clinical data hinder confident conclusions despite positive findings in animal models.

#### GLP-2

A hormone consisting of 33 amino acids, GLP-2 is encoded by a section of the proglucagon gene that is located closely to the sequence that encodes GLP-1. Following its secretion from gut endocrine cells, GLP-2 promotes the absorption of nutrients through distinct mechanisms of action [66]. Additionally, GLP-2 increases the barrier function of the gut epithelium and regulates gastric motility, gastric acid secretion, and intestinal hexose transport [66, 67]. In healthy subjects, subcutaneous injections of GLP-2 elicit a dose-related decrease in CTX-1 (a bone resorption marker), which has sparked suggestions for the use of GLP-2 as a potential osteoporosis treatment [68]. Despite a described effect of GLP-2 on osteoclast activity, the GLP-2 receptor has not been identified in human osteoclasts or any other bone-related cell type [69], except for immature human osteoblast cell lines MG-63 and TE-85 [69]. In a clinical study published by Gottschalck et al. exogenous GLP-2 administration decreased serum and urinary markers of bone resorption and increased hip BMD in postmenopausal women and spine BMD in patients with short bowel syndrome [70]. No studies have reported the effects of GLP-2 on bone remodeling in mice [50].

More recently, unimolecular incretin agonists have been engineered by Gobron et al. [71]. The authors developed a series of unimolecular dual GIP/GLP-2 analogs with the first-in-class molecule GL-0001 being capable of enhancing collagen maturity, improving bone biomechanical response, and increasing resistance to fractures in vivo. The study's emphasis on targeting bone material properties rather than BMD alone was innovative and different from conventional methods for treating bone fragility [71].

A randomized, double-blind, placebo-controlled, crossover study evaluated bone markers of formation and resorption in 17 overweight or obese men without T2DM who received sequence infusions of GIP alone, GLP-1 alone, a combination of GIP and GLP-1, and placebo [72]. The results showed that the combination of GIP and GLP-1 had an additive effect by suppressing bone resorption markers (74, Fig. 3, Table 1). Similar to GIP, GLP-1 led to a notable suppression of the bone resorption marker CTX-1. The reduction in CTX-1 was greater when both incretin hormones (GLP-1 and GIP) were administered together, compared with each hormone administered alone. Notably, P1NP levels were unaffected by the interventions. The study's results suggest that both GLP-1 and GIP suppress bone resorption. Future research on dual-receptor agonists may help shed light on their potential benefits in bone health.

In summary, GLP-2 has a significant inhibitory effect on bone resorption with minimal impact on bone formation, resulting in increased BMD. Studies suggest that only supraphysiological doses of exogenous GLP-2 effectively reduce bone resorption (CTX-1). However, the specific mechanism by which GLP-2 influences bone metabolism remains unknown. It is uncertain whether GLP-2 acts directly on bone cells or if its effects are mediated indirectly, possibly involving other intestinal factors ([50], Fig. 3).

#### NPY and PYY

A part of the pancreatic polypeptide family, NPY is a 36-amino acid peptide. It is primarily produced and expressed in the central and peripheral nervous system, with significant expression in the hypothalamus. Notably, NPY plays a significant role in various physiological processes, including the regulation of appetite, stress responses, and control of blood pressure. Its widespread distribution in the nervous system underscores its importance in modulating a wide range of physiological functions [73]. Expression of NPY by osteoblasts, osteoclasts, osteocytes, chondrocytes, and adipose tissue has recently been described [74]. This action on bone metabolism caught the attention of several researchers in the area and has become a hot topic in recent years. Additionally, NPY acts as a multifunctional neurotransmitter and neuromodulator through a family of G-protein coupled receptors known as Y receptors [73].

There are five known subtypes of Y receptors, namely, Y1R, Y2R, Y4R, Y5R, and Y6R. The interplay between these receptors and NPY in the context of bone mass regulation, an area of active research, highlights the complex role of NPY in the body's regulatory systems [75]. Of these receptor subtypes, Y1R and Y2R are particularly involved in modulating bone mass, but they do so through different mechanisms and at different sites. The Y1R subtype is primarily expressed in osteoblasts. A Y1R germline deletion results in elevated osteoblast activity and mineral apposition rate, together with increased formation of highly multinucleated osteoclasts and enhanced surface area, demonstrating a negative role of Y1R on bone mass maintenance [76]. When truncated by DPP-4, NPY has a half-life of 2 to 3 min, after which it loses the ability to bind to the Y1R [76]**.** The Y2R subtype, on the other hand, is expressed in sympathetic nerve fibers that innervate bone tissue and can influence bone remodeling by regulating the sympathetic nervous system's activity. Mice with Y2R knockout in the hypothalamus have increased osteoblastic activity, mineralization rate, and bone mass, indicating that Y2R normally plays a catabolic role in stimulating cortical and cancellous bone formation [77].

In postmenopausal osteoporosis, NPY is upregulated in bone tissue. This upregulation of NPY may contribute to the bone loss seen after menopause [78, 79]. In osteoporosis associated with glucocorticoid-induced bone loss, NPY mRNA expression and protein concentration are elevated [79]. This elevation of NPY has been associated with a significant reduction in BMD and bone microstructure, which suggests that NPY may contribute to the negative effects of glucocorticoids on bone health [79].

The pancreatic peptide YY (PYY), a member of the pancreatic polypeptide family, is another gastrointestinal peptide released after food ingestion. It is cosecreted along with GLP-1 e GLP-2 and is considered a physiological DPP-4 substrate. Upon secretion, PYY is released as a peptide consisting of 36 amino acids known as PYY 1–36. After secretion, PYY 1–36 is metabolized by DPP-4 to form PYY 3–36 [75]. Interestingly, PYY 1–36 binds to Y1R, Y2R, and Y5R, whereas PYY 3–36 has a high affinity for Y2R [80]. A possible action of PYY is a catabolic effect on bone [50]. In certain conditions characterized by low bone mass in humans, PYY is upregulated. An inverse correlation is observed between plasma PYY and BMD in populations with weight gain and obesity (decreased PYY and increased BMD) and in weight loss scenarios (increased PYY and decreased BMD), as observed in patients with anorexia and amenorrheic athletes [81].

The PYY concentration increases significantly after Roux-en-Y gastric bypass (RYGB), potentially contributing to the notable bone loss observed after this procedure. This bone loss exceeds what can be attributed solely to the substantial weight reduction associated with RYGB. Concurrently, there is a rise in CTX-1 levels following gastric bypass, directly correlating with the alterations in PYY levels. Patients undergoing weight loss after gastric banding demonstrate no significant changes in either PYY or CTX-1 concentrations. This discrepancy between the effects of RYGB and gastric banding on PYY and CTX-1 supports a connection between PYY and bone markers, particularly in the context of bone health markers after bariatric surgery [82], (Table 1).

In summary, the formation of PYY is decreased by DPP-4 inhibition. [76, 77, 81, 83]. Both PYY and NPY share the same receptors (Y receptors, notably Y1R and Y2R), which regulate bone mass [80]. Activation of Y1R results in osteoclast formation, negatively impacting bone maintenance, while activation of Y2R influences bone remodeling by modulating the activity of the sympathetic nervous system.

### Adipokines: adiponectin and leptin and their relationship with DPP-4

Adiponectin, another DPP-4 substrate hormone, is related to energy metabolism and is primarily secreted by brown adipose tissue and bone marrow adipose tissue [84]. It holds a significant role in obesity, glucose, lipid metabolism, and cardiovascular disease [84]. Evidence has shown a negative correlation between DPP-4 activity and circulating adiponectin levels in lean and obese subjects [85].

In relation to bone metabolism, receptors for adiponectin have been described in osteoblasts and osteoclast*s* [84]. However, the involvement of adiponectin in bone homeostasis is intricate and influenced by various adiponectin isoforms and adiponectin receptor subtypes, with conflicting findings between animal and human studies. Based on gathered evidence, DPP-4 may reduce the putative positive impact of adiponectin on bone mass [85].

Rats with DPP-4 deficiency display enhanced adiponectin levels along with attenuated adipose tissue inflammation and insulin resistance [86]. Mice lacking adiponectin exhibit reduced bone mass and increased adiposity. Additionally, adiponectin suppresses essential signaling pathways, including nuclear factor-kB (NF-kB) and p38, which are crucial for osteoclast formation [87].

Although preclinical data generally suggest a positive impact of adiponectin on bone homeostasis through the reduction in osteoclast activity and the increase in osteoblastic differentiation, clinical studies present conflicting results. Some studies indicate an inverse correlation between adiponectin levels and BMD [88–91], particularly among individuals with osteoporosis. This possibly occurs by stimulation of the RANKL pathway and inhibition of production of the decoy receptor for RANKL/osteoprotegerin, which differs from findings from preclinical studies [92]. Reinforcing this trend, a recent case–control study emphasized a robust inverse connection between adiponectin and T scores in women with osteoporosis and osteopenia [93]. Additionally, a large prospective study introduced a notable sex-specific aspect to the association between adiponectin and bone, revealing that high adiponectin levels were associated with a greater risk of fractures in men, independent of body composition and BMD, while no such association was observed in women [94]**.** This suggests that adiponectin may function as a unique predictor of increased fracture risk specifically in the male sex. Finally, a systematic review and meta-analysis of randomized controlled trials has shown that the use of DPP-4 inhibitors leads to elevated plasma concentrations of adiponectin [95].

In short, the results of the association between adiponectin and bone metabolism are quite discrepant between preclinical and clinical studies. More studies are currently needed to improve the understanding of the bone effects of this hormone ([88–91, 93, 96–98], Table 1).

Leptin, another adipokine, is not a confirmed substrate for DPP-4 like adiponectin but may have a putative DPP-4 truncation site [99]. Produced by subcutaneous fat, skeletal muscle, bone marrow adipocytes, and chondrocytes [100], leptin exerts a dual effect on bone tissue; it can centrally inhibit bone formation by binding to leptin receptors in the hypothalamus or locally promote bone formation and inhibit bone resorption by binding to receptors expressed on the surface of osteoblasts [100]. Leptin may also suppress RANKL production and increase osteoprotegerin levels [101]. Most clinical studies on leptin administration have been conducted in women with hypothalamic amenorrhea, which is known to be associated with reduced leptin levels. Two randomized controlled trials in women with hypothalamic amenorrhea have shown conflicting results: one indicated an increase in osteocalcin and N-telopeptides of type 1 collagen (NTX) but no change in BMD [102], while the other revealed increased spine BMD in lean women with hypoleptinemia [101, 103, 104].

In summary, studies evaluating the associations between leptin and BMD in humans have shown mixed results [106]. Large prospective longitudinal studies, including clinical trials, are needed to comprehensively explore the regulatory impact of leptin on bone and its potential implications for fracture risk (110, Table 1).

## Inhibition oF DPP-4 activity and bone metabolism

Some studies have shown that greater DPP-4 levels or activity correlate with decreased BMD as well as increased bone resorption markers, risk of fractures, and inflammatory markers (*e.g.*, IL-6 and high-sensitivity CRP, 6). This evidence suggests that DPP-4 may play a significant role in regulating bone health and inflammatory response [2, 5, 6, 8–10].

DPP-4 inhibitors play a significant role in glycemic regulation and improving glycemic control in patients with type 2 diabetes mellitus (T2DM). The incidence of hypoglycemia is relatively low due to their mechanism of action. This is particularly important because hypoglycemia is a common cause of falls and subsequent fractures, especially in older adults and those with longer-standing diabetes. Therefore, patients who are more predisposed to fractures can benefit significantly from these medications, making DPP-4 inhibitors a very appealing therapeutic option for the elderly [26]. A retrospective population-based cohort study demonstrated a longitudinal relationship over 2 years between glycated hemoglobin (HbA1c) levels and increased fracture risk among individuals with T2DM. After adjusting for covariates, poor glycemic control in T2DM patients was associated with a 29% higher risk of fractures compared to those with adequate glycemic control. Treatment with metformin and DPP-4 inhibitors was associated with a reduced risk of fractures overall [129].

Beyond their primary role in improving glycemic control in patients with T2DM, DPP-4 inhibitors also demonstrate different effects on bone metabolism [107], *e.g.*, through actions on DPP-4 substrates and adipokines [99]. Most randomized controlled trials and observational and clinical studies have demonstrated that DPP-4 inhibitors are safe in regard to bone and may decrease the risk of fractures in patients with T2DM [37]. Although the effect on glucose levels is a class effect of all DPP-4 inhibitors, some of them have different and discrepant actions on bone metabolism. Vildagliptin appears to have a neutral effect, while saxagliptin has a negative effect on bone, increasing osteoclastic activity and decreasing osteocytic and osteoblastic activity in the femur in preclinical studies [108, 109, 109]. Some clinical studies have shown detrimental effects of DPP-4 inhibitors on bone [13, 16, 18], with one study showing no effects [110]. Having an active metabolite is a unique feature of saxagliptin compared with other DPP-4 inhibitors. Whether this distinct property of saxagliptin could interact with pathways of bone metabolism and bone turnover, thus having a relatively negative impact on bone mass or strength, needs to be elucidated. Sitagliptin and linagliptin are the strongest DPP-4 inhibitors with the greatest potential to improve bone metabolism, as demonstrated in preclinical and clinical studies [107].

Another way in which DPP-4 inhibitors may affect bone metabolism is through a pathway linked to 25(OH)-D levels (Vitamin D, [136]). DPP-4 inhibitors significantly raise 25(OH)-D levels in serum, promoting bone growth and remodeling [136]. These effects are mediated through several mechanisms: DPP-4 modulates inflammation in adipose tissue, a major site of vitamin D accumulation and action [22]. In diabetic mice, DPP-4 inhibition with sitagliptin reduces adipose tissue inflammation, potentially enhancing vitamin D activation and release from adipocytes into the bloodstream [22]. Additionally, DPP-4 inhibitors such as linagliptin inhibit the receptor for Advanced glycation end products (RAGE) expression in keratinocytes, which can facilitate local vitamin D production by preventing interference from accumulated Advanced glycation end products (AGEs, 142).

Tables 2, 3, 4  and 5 summarize the main preclinical and clinical studies on the effects of DPP-4 inhibitors on bone metabolism.

**Table 2:** Preclinical studies on the effects of dipeptidyl peptidase 4 (DPP-4) inhibitors on bone metabolism

Studies	Animal types	Methodology	Main results
Kyle et al. 2011 [111]	Female/male rats (C57BL/6 lineage) without T2DM	Pioglitazone vs. sitagliptin vs. genetic DPP-4 OVX vs. no-OVX Inactivation Treatment:12 weeks	**BMD:** Sitagliptin treatment significantly improved vertebral volumetric BMD in female mice (no-OVX) **Bone quality**: Sitagliptin significantly improved trabecular architecture and reduced trabecular separation in female mice (not OVX)
Cusick et al. 2013 [112]	Ovariectomized (OVX) Sprague–Dawley rats without T2DM	Sitagliptin (100, 300, or 500 mg/kg/day) vs. control group Treatment:12 weeks	**BMD:** generally did not differ significantly between OVX-sitagliptin-treated animals and OVX-vehicle controls Significantly lower BMD loss in lumbar vertebrae with Increasing sitagliptin dose
Glorie et al. 2014[113]	Male Wistar rats T2DM	Sitagliptin vs control group Sitagliptin (100, 300, or 500 mg/kg/day Treatment:12 weeks	**Bone quality:** reduction in trabecular loss and prevention of stagnation in vertical bone growth in rats treated with the DPP4 inhibitor compared with placebo **Bone strength:** biomechanical test demonstrated an increase in maximum load until fracture in T2DM animals treated with sitagliptin vs. untreated ones
Gallagher et al. 2014 [114]	Male wild-type (WT) and diabetic muscle-lysine-arginine (MKR)	MK-0626 4 g/kg vs. control vs. pioglitazone	**Bone quality:** MK-0626 has neutral effects on cortical and trabecular bone
Eom et al. 2016 [115]	Male Zucker rats with T2DM	Vildagliptin 10 mg/kg vs. pioglitazone Treatment: 4 weeks	**Bone quality**: Improvement in bone density and microarchitecture in rats treated with vildagliptin compared with pioglitazone
Sbaraglini et al. 2016 [109]	Male Sprague–Dawley rats with T2DM	Saxagliptin	**Bone quality:** Treatment-induced significant decrease in femoral osteocytic and osteoblastic density of metaphyseal trabecular bone and in the average height of the proximal cartilage growth plate, and increase in osteoclastic tartrate-resistant acid phosphatase (TRAP) activity in the primary spongiosa
Mansur et al. 2018 [116]	Male Swiss mice with T2DM	Sitagliptin 50 mg/kg vs. control Treatment: 3 weeks	**Bone quality:** Increased osteoid perimeter vs. control. No changes in trabecular or cortical microarchitectures. Ameliorations in bone strength at the organ and tissue level in the sitagliptin group
Charoenphandhu et al. 2018 [108]	Male rats	Vildagliptin 3 mg/kg Vs. control group Treatment:12 weeks	**Bone quality:** greater trabecular bone volume and number, less trabecular separation. No change in trabecular thickness, osteocyte lacunar area, or cortical thickness in the vildagliptin-treated group
Kanda et al. 2020[117]	Male leptin-deficient mice with T2DM	Linagliptin 3 or 30 mg/kg vs. control Treatment: 12 weeks	**Bone quality:** Trabecular bone volume increased and decreased bone resorption with 30 mg/kg dose
NIRWAN et al. 2022 [118]	Male rats (C57BL6 lineage) with T2DM	Linagliptin (10 mg /kg) as monotherapy or in combination with metformin Treatment: 4 weeks	Improvement in bone density and bone microarchitecture in rats treated with linagliptin, which was not observed in isolated treatment with metformin alone
Abdi et al. 2023 [119]	Male Wistar rats	Sitagiptin10 mg/kg or exenatide Treatment: 5 weeks	**Bone quality:** Histomorphometric results on both drugs showed reduced bone resorption. Diminished bending strength in sitagliptin-treated rats

*BMD* bone mineral density, *OVX* ovariectomized

**Table 3:** Meta-analysis and systematic reviews on the effects of dipeptidyl peptidase 4 (DPP-4) inhibitors on bone metabolism

Studies	Study type and population	Methodology	Main results
Monami et al. 2011[14]	Meta-analysis: 28 RCTs Patients with T2DM Men and women Age range: 55–71 years No data about menopause	N = 11,880 DPP-4 inhibitor vs. placebo (N = 9,175) and active comparators (exenatide, liraglutide, thiazolidinediones, metformin, sulfonylureas, acarbose)	**Fracture risk:** Reduced with DPP-4 inhibitors (OR 0.60, 95% CI 0.37–0.99) compared with placebo or other treatments **Limitations**: Bone fractures were not the main endpoints and were reported only as adverse events; the studies had a short follow-up duration
Fu et al. 2016 [135]	Meta-analysis of 62 RCTs Patients with T2DM Men and women Age range: 49.7–74.9 years No data on menopause	N = 62,606 DPP-4 inhibitor users (alogliptin, saxagliptin, linagliptin, sitagliptin, anagliptin, and vildagliptin) versus placebo versus active comparators (albiglutide, canagliflozin, empagliflozin, glipizide, glimepiride, metformin, voglibose, or thiazolidinediones)	**Fracture risk:** No differences in risk of fracture (RR 0.95, 95% CI 0.83–1.10) even in subgroups using different types of DPP-4 inhibitors; different types of control; different follow-up durations **Limitations:** Bone fractures were not the main endpoints and were reported only as adverse events; the studies had short follow-up duration
Mamza et al. 2016 [17]	Systematic review and meta-analysis of 51 RCTs Patients with T2DM Mean age: 57.5 ± 5.4 years Women: 47% No data on menopause	N = 36,402 DPP-4 inhibitor (alogliptin, saxagliptin, sitagliptin, vildagliptin) versus placebo versus active comparators (GLP-1 receptor agonists, canagliflozin, glipizide, metformin, voglibose, or thiazolidinediones)	**Fracture risk:** No significant association of fracture events with the use of DPP-4 inhibitors when compared with placebo (OR 0.82, 95% CI 0.57–1.16) or against an active comparator (OR 1.59, 95% CI 0.91–2.80) **Limitations:** Included trials with follow-up too short for detection of fractures
Yang et al. 2017 [16]	Systematic review and meta-analysis of 75 RCTs Patients with T2DM Mean age: 57.8 ± 5.5 years No data on menopause	N = 70,207 Users of DPP-4 inhibitors (alogliptin, linagliptin, saxagliptin, sitagliptin, and vildagliptin) versus placebo versus active comparators (sulfonylureas, GLP-1 receptor agonists, metformin, thiazolidinediones, SGLT2is)	**Fracture risk:** Alogliptin tended to decrease the risk compared with placebo (OR 0.51, 95% CI 0.29–0.88) and had a lower risk compared with linagliptin (OR 0.45, 95% CI 0.20–0.99) and saxagliptin (OR 0.46, 95% CI 0.25–0.84). The risk was higher with saxagliptin versus sitagliptin (OR 1.90, 95% CI 1.04–3.47) **Limitations:** The duration of some trials was too short for long-term data on the effects of the drugs on fracture
Hidayat et al. 2019 [133]	Systematic review and meta-analysis of 18 RCTs Patients with T2DM Age range: 50–73 years No data on menopause	Association between the use of DPP-4 inhibitors, GLP-1 receptor agonists, or SGLT-2is and the risk of fracture compared with controls and active comparators	**Fracture risk:** Use of DPP-4 inhibitors (RR 0.83, 95% CI 0.60–1.14) was not associated with risk of fracture compared with controls or active comparators **Limitations:** Fracture events were only rarely confirmed by radiographic imaging; most of the included RCTs were conducted in Western countries, limiting extrapolation of the data to Asian populations
Zhang et al. 2021 [13]	Network meta-analysis of 117 RCTs T2DM Age: not available No data on menopause	N = 221,364 Head-to-head comparison of antidiabetic drugs (SGLT2is, DPP-4 inhibitors, GLP-1receptor agonists, meglitinides, alpha-glucosidase inhibitors, thiazolidinediones, biguanides, insulin, and sulfonylureas) on fracture risk	**Fracture risk:** Increased with omarigliptin (RR 1.33, 95% CI 0.21–8.24), sitagliptin (RR 1.29, 95% CI 0.27–6.47), vildagliptin (RR 1.17, 95% CI 0.23–6.16), and saxagliptin (RR 2.04, 95% CI 0.38–12.09) and decreased with linagliptin (RR 0.9, 95% CI 0.18–4.66) and alogliptin (RR 0.76, 95% CI 0.12–4.87). However, only trelagliptin (RR 3.51, 95% CI 1.58–13.70) increased the risk of fracture significantly
Kong et al. 2021 [134]	Meta-analysis of 112 RCTs Patients with T2DM Age range: 49.4–71.6 years No data about menopause	N = 111,539 Incretins (alogliptin, linagliptin, saxagliptin, sitagliptin vildagliptin, omarigliptin and anagliptin, dulaglutide, exenatide, lixisenatide, and liraglutide) users versus placebo versus active comparators	**Fracture risk**: Reduced with sitagliptin (OR 0.495, 95% CI 0.304–0.806) and liraglutide 1.8 mg (OR 0.621, 95% 0.413–0.933)
Chai et al. 2022 [135]	Systematic review and network meta-analysis of 177 RCTs Age range: 53–74.9 years No data about menopause	N = 165,081 DPP-4 inhibitor versus placebo versus active comparators (GLP-1 analogs, SGLT-2i, sulfonylureas, insulin, metformin, alpha-glucosidase inhibitor, or thiazolidinediones)	**Fracture risk:** Not increased with DPP-4 inhibitors compared with active comparators or placebo **Limitations**: Most of the included RCTs were conducted in Western countries; limited data in Asians
Huang et al. 2024 [12]	Meta-analysis of 10 RCTs Patients with T2DM Age range: 54–68 years No data on menopause	N = 214,541 DPP-4 inhibitors (sitagliptin, linagliptin, vildagliptin, saxagliptin, gemigliptin, teneligliptin, alogliptin, and anagliptin)	**Fracture risk:** Not evaluated Use of DPP-4 inhibitors increased BMD (SMD 0.15, 95% CI 0.03–0.26) and reduced the risk of osteoporosis (SMD 0.81, 95% CI 0.72–0.9)

*CI* confidence interval, *GLP-1* glucagon-like peptide 1, *RCT* randomized controlled trial, *OR* odds ratio, *RR* risk ratio, *SGLT2i* sodium-glucose cotransporter 2 inhibitor, *SMD* standardized mean difference, *T2DM* type 2 diabetes mellitus

**Table 4:** Randomized controlled trials on the effects of dipeptidyl peptidase 4 (DPP-4) inhibitors on bone metabolism

Study	Study type and population	Methodology	Main results
Mosenzon et al. 2015 [110]	RCT T2DM Saxagliptin Assessment of Vascular Outcomes Recorded in Patients with Diabetes Mellitus–Thrombolysis in Myocardial Infarction 53 (SAVOR-TIMI 53) trial Median age: 66 ± 12 years Women:49.1%	N = 8,280 Saxagliptin vs. placebo	**Fracture risk**: no significant difference between saxagliptin and placebo (HR 1.00, 95% CI 0.83–0.19)
Josse et al. 2017 [33]	RCT T2DM Subgroup analysis of the randomized trial Evaluating Cardiovascular Outcomes with Sitagliptin (TECOS) Mean Age: 65 ± 8 years	N = 14,671 Sitagliptin vs. placebo	**Fracture risk:** no significant difference between sitagliptin and placebo (95% CI aHR 1.03, p = 0.745)
Espeland et al. 2020 [130]	RCT T2DM Subgroup analysis of the randomized trial CAROLINA Mean Age: 64 years	N = 6,033 Linagliptin vs. glimepiride	**Fracture risk:** lower fracture risk with linagliptin vs. glimepiride (HR 0.76, 95% CI 0.63–0.93)
Ha et al. 2021 [131]	Prospective, multicenter, open-label, comparative trial T2DM Mean Age: 66.5 ± 7.1 years 100% women Postmenopausal	N = 264 Subjects who had previously been treated with metformin monotherapy or combination metformin/sulfonylurea were divided into four groups: Group 1, continued metformin monotherapy or combination metformin/sulfonylurea; Group 2, addition of thiazolidinedione (TZD); Group 3, addition of DPP-4 inhibitor (gemigliptin); and Group 4, addition of SGLT2i	**Fracture risk:** No data on fractures Bone loss was not significantly associated with DPP-4 inhibitor use

*aHR* adjusted hazard ratio, *CI* confidence interval, *HR* hazard ratio, *RCT* randomized controlled trial, *SGLT-2i* sodium-glucose cotransporter-2 inhibitor, *T2DM* type 2 diabetes mellitus

**Table 5:** Cohort studies on the effects of dipeptidyl peptidase 4 (DPP-4) inhibitors on bone metabolism

Study	Study type and population	Methodology	Main results
Hirschberg et al. 2014 [18]	Post hoc pooled analysis of 20 RCTs T2DM Age: 81% of the cohort < 65 years Women: 50.9%	N = 9156 Saxagliptin as monotherapy or add-on therapy	**Fracture risk**: Higher with saxagliptin versus control (incidence 1.1 versus 0.6, respectively, OR 1.81, 95% CI 1.04–3.28)
Driessen et al. 2014 [15]	Cohort study T2DM Mean age: 61 ± 21 years Women: 43% The Clinical Practice Research Datalink	N = 216,816 Use of DPP-4 inhibitor versus other antidiabetic drugs (NIAD) or controls*	**Fracture risk**: DPP-4 inhibitor was not associated with risk of any fracture (adjusted HR 0.89, 95% CI 0.71–1.13). No different risk of fracture comparing current DPP-4 inhibitor users versus controls (adjusted HR 0.89, 95% CI 0.71–1.13). No increased risk comparing DPP-4 inhibitor users versus other NIADs (adjusted HR 1.03, 95% CI 0.92–1.15)
Majumdar et al. 2016 [121]	Cohort study T2DM Median age: 52 years Women: 46% The Clinformatics Data Mart Database	N = 72,738 New users of sitagliptin versus nonusers of sitagliptin	**Fracture risk**: sitagliptin was not associated with fracture (adjusted HR 1.1, 95% CI 0.8–1.4)
Choi et al. 2016 [122]	Cohort study T2DM Age: ≥ 50 years	N = 207,558 Metformin + DPP-4 inhibitor initiators versus nonusers of glucose-lowering medications*	**Fracture risk**: Metformin + DPP-4 inhibitor combination was significantly associated with reduced composite fracture risk compared with nonusers of glucose-lowering medications (HR 0.83, p = 0.025)
Wallander et al. 2016 [123]	Cohort study T2DM Mean age: 80.8 ± 8.2 years Women: 58% The Swedish registry “Senior Alert” (2008–2014	N = 79,159 Sitagliptin versus nonusers of sitagliptin	**Fracture risk**: sitagliptin was not associated with hip fracture in users of this medication compared with nonusers (HR 0.85, 95% CI 0.64–1.12)
Dombrovski et al. 2017 [11]	Cohort study T2DM Mean age: 61.6 ± 11.1 years Women:40.4% The Disease Analyzer database (IMS HEALTH)	N = 1.262 DPP-4 inhibitors ever users versus never users *(1:1)	Fracture risk: DPP-4 inhibitor decreased the risk of bone fractures (HR 0.67, 95% CI 0.54–0.84)
Driessen et al. 2014[15]	Cohort study T2DM Mean age: DPP-4 inhibitors users, 59.7 ± 12.4; non-insulin antidiabetic drugs, 61.5 ± 16.1 years The Clinical Practice Research Datalink	N = 328,254 Current users of DPP-4 inhibitors versus current users of NIAD (excluding GLP-1 receptor agonists)*	**Fracture risk***:* Current use of DPP-4 inhibitor was not associated with risk of any fracture compared with current nonuse of insulin or antidiabetic drug (adjusted HR 0.99, 95% CI 0.93–1.06)
Losada et al. 2018 [124]	Case–control study T2DM Mean age: 72.90 ± 11.40 years (cases) and 72.86 ± 11.36 years (controls) System for Research Development in Primary Care	N = 12,277 DPP-4 inhibitors versus other antidiabetic drugs, including insulin	**Fracture risk**: No increase in fracture risk with DPP-4 inhibitors versus other antidiabetic drugs, including insulin (OR 1.23, 95% CI 0.68–2.20)
Hou et al. 2018 [125]	Cohort study T2DM Mean age: 53.86 ± 12.33 years (non-DPP-4 inhibitor users) and 54.00 ± 12.72 years (DPP-4 inhibitor users) Women: 46% Medical claims data of the Longitudinal Cohort of Diabetes Patients	N = 3,996 DPP-4 inhibitor (saxagliptin, linagliptin, and sitagliptin) as a second-line antidiabetic drug versus same number of matched DPP-4 inhibitor nonusers	**Fracture risk**: decreased with DPP-4 inhibitor (HR 0.86, 95% CI 0.74–0.99)
Gamble et al. 2018 [125]	Cohort study T2DM Mean age: 57.1 ± 12.1 years (DPP-4 users) and 59.3 ± 13.5 years (controls) Women: 40% UK-based Clinical Practice Research Datalink	N = 34,629 New users of DPP-4 inhibitors versus new users of sulfonylureas *	**Fracture risk**: No significant difference between treatments (adjusted HR 0.80, 95% CI 0.51–1.24)
Ustulin et al. 2019 [126]	Cohort study T2DM Age: ≥ 50 years Women: 34% National Health Insurance Service–National Sample Cohort	N = 11,164 DPP-4 inhibitor users versus nonusers versus controls*	**Fracture risk**: No significant difference in fracture risk (adjusted HR 0.80, 95% CI 0.51–1.24). In a secondary analysis, DPP-4 inhibitors were not associated with a difference in fracture risk compared with insulin (adjusted HR 0.91, 95% CI 0.40–2.09) but were associated with a lower fracture risk versus thiazolidinediones (adjusted HR 0.47, 95% CI 0.26–0.83)
Chang et al. 2022 [127]	Cohort study T2DM Mean Age: 66 ± 11.9 years Women: 48.2% Taiwan’s Longitudinal Health Insurance Database	N = 31,695 DPP-4 inhibitor users versus nonusers*	**Fracture risk:** No data on fractures. The risk of all-cause osteoporosis was significantly lower in DPP-4 inhibitor users versus nonusers (adjusted HR 0.616, 95% CI 0.358–0.961)
Al-Mashadi et al. 2022 [128]	Cohort study T2DM Mean age: 56.6 ± 12.0 years (GLP-1 group) and 63.6 ± 12.4 years (DPP-4 inhibitor group) Women: 42% Danish National Health Registries	N = 32,266 Risk of MOF for treatment with GLP-1 receptor agonist versus DPP-4 inhibitor as add-on therapies to metformin*	**Fracture risk**: No significant difference in risk between treatment with GLP-1 receptor agonist and DPP-4 inhibitor (HR 0.86, 95% CI 0.73–1.03)
Wang et al. 2022 [129]	Cohort study T2DM Age: 66.04 ± 7.27 years Women: 49.9% OptumLabs Data Warehouse	N = 15,439 DPP-4 inhibitor users versus other antidiabetic drug users, including insulin	**Fracture risk**: DPP-4 inhibitor use was associated with reduced all-fracture risk compared with other treatments (HR 0.93, 95%CI 0.88–0.98). All clinical fractures were evaluated, including typical fracture types attributed to T2DM, such as hip and vertebral ones

*aHR* adjusted hazard ratio, *CI* confidence interval, *HR* hazard ratio, *MOF* major osteoporotic fracture, *N* number of participants, *NIAD* noninsulin antidiabetic drugs, *OR* odds ratio,*RCT* randomized controlled trial, *T2DM* type 2 diabetes mellitus. *Type of DPP-4 inhibitor unspecified

## Conclusions

This review provides insights into the influence of DPP-4 on bone metabolism and delineates the potential mechanisms of the interaction between DPP-4 and bone (Fig. 2). Although the direct inhibition of DPP-4 activity does not seem to regulate bone remodeling, the impact of DPP-4 on bone metabolism is indirect, involving the modulation of DPP-4 substrates and inflammation within the bone microenvironment. These findings suggest that increased DPP-4 activity could indirectly foster bone resorption while hindering bone formation, thereby elevating the risk of osteoporosis. This opens up avenues for a novel understanding of the role of DPP-4 in the mechanisms underlying osteoporosis.

Notably, DPP-4 inhibitors appear to be safe regarding the risk of fractures, as they tend to decrease this risk, but more clinical trials are needed to explore the effects of DPP-4 inhibitors in other populations beyond T2DM. This is particularly important if these inhibitors are shown to affect bone metabolism through independent mechanisms beyond glucose control. The conflicting data in some clinical studies may be explained by various factors: [1] most cohort studies lacked individual validation of fractures as primary outcomes, [2] the studies had short follow-up duration and [3] did not consider important risk factors for osteoporosis such as BMD (even though BMD is not a good method for diagnosing osteoporosis in T2DM), and [4] some studies included medications that affect fracture risk, such as corticosteroids or antidepressants. Most studies did not identify postmenopausal women separately, and some cohorts had more men than women. Although the use of DPP-4 inhibitors is associated with increased bone formation, their effects are more associated with mechanisms related to the suppression of bone resorption. Thus, the potential positive effect of DPP-4 inhibitors on osteoporosis and fractures may be more apparent in postmenopausal women because of higher bone remodeling. Another limitation of the studies was the use of different comparators ranging from placebo controls to other medications for T2DM (including insulin), which have different effects on bone tissue and were included in only one group, misleading the interpretation. Other information lacking in some studies was the identification of diabetic complications (retinopathy, nephropathy, neuropathy), which could have affected the choice of insulin or fracture risk. Some studies were conducted in Asia and others only in Europe, precluding the application of the results to populations of different ethnic backgrounds.

The widespread use of DPP-4 inhibitors among patients with T2DM and advanced age, who are more predisposed to osteoporosis, underscores the need for a better understanding of the relationship between DPP-4 enzyme activity, its substrates, pharmacological inhibition, and bone metabolism.
